# Supplementary material for: Virtual reconstruction of the endocranial anatomy of the early Jurassic marine crocodylomorph Pelagosaurus typus (Thalattosuchia)
Source: PeerJ. 2017 Apr 25;5:e3225. doi: 10.7717/peerj.3225 (PMC5407279; doi:10.7717/peerj.3225)
Supplement: Figure S2 — Note, best viewed with CAD Optimized Lighting. [file peerj-05-3225-s002.pdf]

Gavialis gangeticus (Pierce et al., 2017)
